# Immuno-Imaging to Predict Treatment Response in Infection, Inflammation and Oncology

**DOI:** 10.3390/jcm8050681

**Published:** 2019-05-14

**Authors:** Alberto Signore, Chiara Lauri, Sveva Auletta, Kelly Anzola, Filippo Galli, Massimiliano Casali, Annibale Versari, Andor W.J.M. Glaudemans

**Affiliations:** 1 Nuclear Medicine Unit, Department of Medical-Surgical Sciences and of Translational Medicine, “Sapienza” University of Rome, 00161 Rome, Italy; chialau84@hotmail.it (C.L.); sveva.auletta@hotmail.it (S.A.); filippo.galli@hotmail.com (F.G.); 2 Department of Nuclear Medicine and Molecular Imaging, University of Groningen, University Medical Center Groningen, 9700 Groningen, The Netherlands; lkanzola@gmail.com (K.A.) a.w.j.m.glaudemans@umcg.nl (A.W.J.M.G.); 3 Nuclear Medicine Department, Clinica Colsanitas, Bogotà 111121, Colombia; 4 Nuclear Medicine Unit, Oncology and High Technology Department, S. Maria Nuova Hospital, AUSL-IRCCS of Reggio Emilia, 42122 Reggio Emilia, Italy; massimiliano.casali@asmn.re.it (M.C.); annibale.versari@asmn.re.it (A.V.)

**Keywords:** infection, inflammation, tumour immunology, therapy follow-up, personalised medicine, tumour microenvironment

## Abstract

Background: Molecular nuclear medicine plays a pivotal role for diagnosis in a preclinical phase, in genetically susceptible patients, for radio-guided surgery, for disease relapse evaluation, and for therapy decision-making and follow-up. This is possible thanks to the development of new radiopharmaceuticals to target specific biomarkers of infection, inflammation and tumour immunology. Methods: In this review, we describe the use of specific radiopharmaceuticals for infectious and inflammatory diseases with the aim of fast and accurate diagnosis and treatment follow-up. Furthermore, we focus on specific oncological indications with an emphasis on tumour immunology and visualizing the tumour environment. Results: Molecular nuclear medicine imaging techniques get a foothold in the diagnosis of a variety of infectious and inflammatory diseases, such as bacterial and fungal infections, rheumatoid arthritis, and large vessel vasculitis, but also for treatment response in cancer immunotherapy. Conclusion: Several specific radiopharmaceuticals can be used to improve diagnosis and staging, but also for therapy decision-making and follow-up in infectious, inflammatory and oncological diseases where immune cells are involved. The identification of these cell subpopulations by nuclear medicine techniques would provide personalized medicine for these patients, avoiding side effects and improving therapeutic approaches.

## 1. Introduction

Nuclear medicine (NM) techniques provide non-invasively functional characterization of diseases that allow a precise and early diagnosis. Also, molecular imaging provides the visualization of cells and molecules that are involved in the process to guide clinicians to choose the best therapy and follow its efficacy for each patient, thereby improving therapy response and avoiding high costs for patient management.

The implementation of hybrid imaging cameras with high sensitivity and the increased development of new radiopharmaceuticals have made possible the targeting of specific molecules and cells expressed in the lesions that are of clinical importance for therapy decision making and follow-up. Furthermore, considering that available radiopharmaceuticals recognise specific target molecules, this specificity could be exploited for therapeutic outcomes by changing diagnostic isotopes with therapeutic ones.

Several radiolabelled molecules such as peptides, cytokines, monoclonal antibodies (MoAbs) and their fragments, and radiolabelled immune cells have been proposed to detect pathological inflammatory sites. They showed promising results, giving the opportunity to move beyond “person-specific” medicine (or personalised medicine), introducing the concept of “lesion-specific” medicine. In particular, in a tumour setting, it becomes more and more clear that different metastases of the same patient may show different biological characteristics thus conditioning a heterogeneous behaviour and response to treatment.

Also, in inflammatory diseases such as rheumatic disorders some lesions could respond to anti-tumor necrosis factor (TNF) and others to anti-CD20 because the different lesions are asynchronous and with different histology even in the same patient.

Therefore, it would be desirable to have several radiolabelled probes available, able to specifically target different molecular pathways involved in the inflammatory process and tumor biology that could be useful to predict and monitor treatment response.

The combination of radiological imaging modalities, such as ultrasound (US), computed tomography (CT) and magnetic resonance imaging (MRI) NM techniques, comprising single photon emission computed tomography (SPECT) and positron emission tomography (PET) might allow us to analyse which target molecules are expressed at which location, to make an early diagnosis, and to decide the best therapy in a diversity of pathologies. Thus, several radiopharmaceuticals may be used in relation to the complex and various pathways involved in the diseases [1,2].

As an example, when using the correct and specific radiopharmaceutical, NM imaging offers the possibility to differentiate septic from aseptic processes through the expression of specific receptors, is able to evaluate the subtypes of T-cells involved in an inflammatory process, may visualize the presence of Gram-positive or Gram-negative bacteria in an infection, or the presence of M1 or M2 macrophages in tissue, and even more.

For these reasons, several efforts have been directed to the development of specific radiopharmaceuticals to image several infectious and inflammatory diseases, as well as several oncological diseases with emphasis on the tumour environment.

In the present review, we describe currently existing NM imaging techniques and radiopharmaceuticals that are able to influence in therapy-decision making in the individual patient and that are able to monitor the therapy efficacy in a wide spectrum of diseases, including infections, inflammation, and tumour immunology.

We performed the research on PubMed and Scopus and selected the most relevant papers/reviews focused on these topics published in the last 15 years.

### 1.1. Infectious Diseases

Radiolabelled white blood cell (WBC) scintigraphy, using both ^99m^Technetium (^99m^Tc) or ^111^Indium (^111^In), is the nuclear medicine gold standard examination for the diagnosis of an infection. Its application covers almost all the field of infective diseases including a lot of indications such as osteomyelitis (OM), and the evaluation of prosthetic joint infections or implantable cardiac devices. However, this procedure is limited by some disadvantages related to the labour extensive process of cellular labelling and the need to acquire images at different time points, which is inconvenient for the patient and leads to logistic problems in a NM department. As an alternative to WBC, we can also use MoAbs such as ^99m^Tc)-besilesomab (Scintimun^®^) to diagnose infections. These radiopharmaceuticals are directed against specific antigens expressed on the surface of granulocytes [3,4,5]. The lack of anatomical landmarks of planar images could represent a limiting factor of both radiolabelled WBC and MoAbs therefore the appeal to SPECT/CT is often mandatory in many specific clinical indications (for example in diabetic foot or vascular graft infection) in order to assess the exact location of the uptake and to evaluate the extent of the infective process.

On the other hand, ^18^F-fluorodeoxyglucose (^18^F-FDG) PET/CT has recently gained a role also in infective and inflammatory disease. Although logistically easier and quicker to perform, this radiopharmaceutical is not specific therefore it is not able to discriminate an infection from an inflammation. However, this modality is very useful in some indications, such as in spondylodiscitis, vasculitis [6] or in a patient with fever of unknown origin or bacteraemia.

Once the infection is stated, it is important to define the aetiology. NM offers a wide variety of radiopharmaceuticals with different targets able to define which kind of infection it is. For this specific purpose, we can use ^99m^Tc-ciprofloxacin (Infecton^®^) that binds to topoisomerase IV and DNA-gyrase expressed by proliferating bacteria targeting Gram positive, Gram negative and anaerobic bacteria [6,7]. Other antibiotics, such as cephalosporins and fluoroquinolones, and other anti-microbial peptides, for example ^99m^Tc-ubiquicidin (UBI), have been tested in pre-clinical animal models. Recently, new probes have been proposed for the detection of fungal infections, such as ^99m^Tc-fluconazole that binds to cytochrome 450 of the microorganism [8]. Viral infections could be potentially detected with the help of an emerging radiopharmaceuticals for PET/CT studies, ^18^F-fluoro-5-ethyl-1beta-D-arabinofuranosyluracil (^18^F-FEAU) that recognizes an enzyme produced by Herpes Simplex virus [9].

Furthermore, NM offers a unique possibility to monitor and evaluate the response to treatment mainly using WBC scintigraphy or ^18^F-FDG PET/CT. Recently, a combination of these two modalities has been proposed through the labelling of leukocytes with ^18^F-FDG for PET studies. However, due to the brief half-life of ^18^F, it is not possible to perform images at a late time point that are often necessary to diagnose an infection.

### 1.2. Inflammatory Diseases

NM offers the possibility to image the presence of T or B-lymphocytes by targeting specific interleukins (IL), chemokines, interferon, natural ligands or antigens expressed by a particular cell. For example, IL2, radiolabelled with both gamma emitters (^99m^Tc, ^123^I) and positron emitters (^18^F), has been extensively studied for imaging activated T-lymphocytes in several chronic inflammatory and autoimmune diseases such as inflammatory bowel disease (IBD), Sjögren syndrome (SS), type 1 diabetes, and thyroiditis [10,11,12,13,14]. The presence of somatostatin receptors (SSTRs) on the surface of activated lymphocytes and fibroblasts in patients affected by rheumatoid arthritis (RA), SS and other chronic inflammatory diseases [15], is the reason why in these diseases radiolabelled somatostatin analogues (SSA) are frequently used for diagnosis and treatment monitoring. Radiopharmaceuticals for SPECT imaging, mainly using SST analogues radiolabelled with ^111^In or ^99m^Tc, have been used in RA, however the appeal to PET/CT with ^68^Gallium (^68^Ga) conjugated octreotide/octreotate peptides (-TOC, -NOC, -TATE) could also be considered, in alternative to gamma-camera studies, since it provides high-quality images with a better resolution compared to SPECT. RA could also be studied using MoAbs and their fragments including anti-TNFα (Infliximab^®^) and anti-CD20 expressed by B-lymphocytes (Rituximab^®^). These molecules can be radiolabelled with ^99m^Tc, ^111^In or ^123^I for SPECT imaging and they could be useful not only in the diagnostic setting, but also for a prognostic evaluation of treatment response [16,17,18,19,20].

The following sections will focus different radiopharmaceuticals useful for diagnosis and therapy monitoring of several infectious and inflammatory diseases.

## 2. Radiolabelled White Blood Cell Scintigraphy for the Diagnosis of Osteomyelitis (OM) and Therapy Follow-Up

OM is an infection that primary involves bone marrow and then extends to cortical bone. Peripheral OM must be distinguished from spondylodiscitis, an infection of the spine, as these two conditions recognize different diagnostic and therapeutic approaches. OM can result from a haematogenous spread of an infection (endogenous type) or from an injury or a surgery (exogenous pathway) [21,22]. Independent of the pathogenesis, OM is a serious condition that should be promptly recognized and treated with an adequate period of specific antibiotics, in order to avoid the evolution from an acute form to a chronic one and to reduce morbidity and mortality [23]. Patients usually report systemic symptoms like fever, fatigue, feeling of illness, nausea and local symptoms, for example pain, reduced mobility of the affected bone and general loss of functionality. Sometimes, in the most striking cases, clear signs of infections can be present like fistula, redness, swelling, and/or purulent secretions [22,23]. In case clear signs are not available, several other diagnostic tests are available. Inflammatory parameters in the blood (C-reactive protein, erythrocyte sedimentation rate) are not specific so the appeal to imaging modality and microbiology is mandatory. Different radiological techniques (US, CT, MRI) are available for the evaluation of OM but they are not always able to discriminate an infection from an inflammation and they usually become only positive in late stages of disease.

NM, on the other hand, offers different strategies that can be used to detect OM in early phases [24]. WBC scintigraphy with in vitro radiolabelled autologous leukocytes, using ^99m^Tc or ^111^In, still remains the NM gold standard as this modality specifically targets the granulocytes and can be used as a surrogate marker of neutrophil-mediated infections [22,25]. For this reason, it is able to discriminate an aseptic inflammation from an infection. Indeed, in the first condition no or exiguous uptake of granulocytes is observed, however, on the contrary, the increased chemotaxis and vascular permeability as a consequence of infective processes, result in an exalted recruitment of granulocytes to the site of infection. The ability specifically to image granulocytes migration through the infected site depends highly from the modality of acquisition and the interpretative criteria adopted [26,27]. The Infection and Inflammation committee of the European Association of Nuclear Medicine (EANM) developed criteria how to correctly label the leukocytes and provided suggestions regarding the acquisition protocols and interpretation criteria of this examination in musculoskeletal infections [28,29,30]. Considering that the recruitment of granulocytes at the site of infection is a dynamic process [31], it is recommended to acquire images at different time points in order to reproduce the physiology. By using the correct strategies, it is possible to discriminate an infection from an aseptic inflammation reaching an accuracy of around 90% that further increases when SPECT/CT is added to planar images for localization of the infectious process (in or outside the bone) [22,32,33,34,35]. Similar outcomes can also be found in another type of peripheral OM, the diabetic foot infection. In a recent systematic review, it emerges that radiolabelled WBC scintigraphy has a sensitivity and a specificity of 92% [36]. Also in the assessment of sternal infections, radiolabelled WBC scan is the NM modality of choice, particularly when SPECT/CT is performed in addition to planar images as it facilitates the evaluation of retrosternal spaces and the extent of the surgical wound thereby making it possible to discriminate between deep and superficial infections [37]. Radiolabelled WBC scintigraphy could also be very useful in order to guide eventual biopsies and for the evaluation of a patient after an adequate antibiotic treatment response thus providing important information on the possibility to stop or to continue the therapy.

When performing WBC scintigraphy, some considerations have to be taken into account. Considering the physiological biodistribution, radiolabelled leukocytes also accumulate into the reticulo-endothelial system, resulting in an increased uptake in expanded healthy bone marrow complicating the interpretation of the scan. So, in some circumstances, a combined WBC/bone marrow scintigraphy could be very useful to distinguish a real infection from physiological bone marrow expansion [38]. For example, this combined imaging modality has been proposed as the best choice for diagnosing periprosthetic knee infections in a recent meta-analysis performed by Verberne and colleagues [39]. Authors obtained an overall specificity and sensitivity equal to 93% and 80%, respectively which was confirmed in another recent review [40].

Another aspect that has to be considered is the decreased accuracy of WBC scintigraphy in chronic infections. This condition is characterized by less neutrophil accumulation and oedema and, consequently, WBC scintigraphy could be less effective. In these situations, radiolabelled anti-granulocyte MoAbs can be used to diagnose OM using similar protocols of acquisition leading to a similar diagnostic accuracy [33,35]. However, the possibility of inducing human murine antibodies (HAMA) in the host limits their use at only one time in the life thus making these radiopharmaceuticals not appropriate for therapy monitoring.

Another issue that is still largely debated is whether ongoing antibiotic therapy could influence the result of the WBC scan [41]. At this moment, there is not enough evidence in literature to draw any definitive conclusions on this topic.

Concluding, although a wide variety of radiopharmaceuticals with different targets are able to image OM, radiolabelled WBC is still the NM gold standard modality since it provides an accurate diagnosis and is useful in therapy decision making in order to guide therapy duration.

## 3. (^18^F)-Fluorodeoxyglucose Positron Emission Tomography/Computed Tomography ((^18^F)-FDG-PET/CT) for Imaging and Monitoring Therapy of Fungal Infections

Invasive fungal infections (IFIs) are often life-threatening since they occur mainly in patients with an already severe underlying disease, such as hematologic or solid malignancies, human immunodeficiency virus/acquired immune deficiency syndrome (HIV/AIDS) or end organ failure. Furthermore, all kind of therapies leading to suppression of the immune system, such as chemotherapy, hematopoietic stem cell transplantation, solid organ transplantation followed by immune suppressive drugs, and immunotherapy also lead to an increase in incidence of IFIs. Most IFIs are caused by *Aspergillus* and *Candida*, but also other types of fungi may occur, such as histoplasmosis, *Cryptococcus* etc.

In patients with IFIs, clinicians often face both a diagnostic and a therapeutic problem. For diagnosis, several possibilities are available such as lung biopsy, broncho-alveolar lavage, galactomannan test, and high-resolution (HR) CT, but all tests have their limitations, and it is often necessary to perform several diagnostic tests in one patient. HR-CT has some pathognomic features, such as the halo sign (ground glass opacity surrounding a nodule or mass) and the air crescent sign (lung cavity filled with air and a round radio-opaque mass), but they are visible mostly in an advanced phase, may be transient, or not visible at all. Furthermore, all aforementioned diagnostic tests focus on pulmonary involvement. But what about the involvement of other organs? Therefore, a non-invasive whole-body imaging test is necessary, and molecular imaging by ^18^F-FDG-PET is able to provide this.

**Figure 1:**
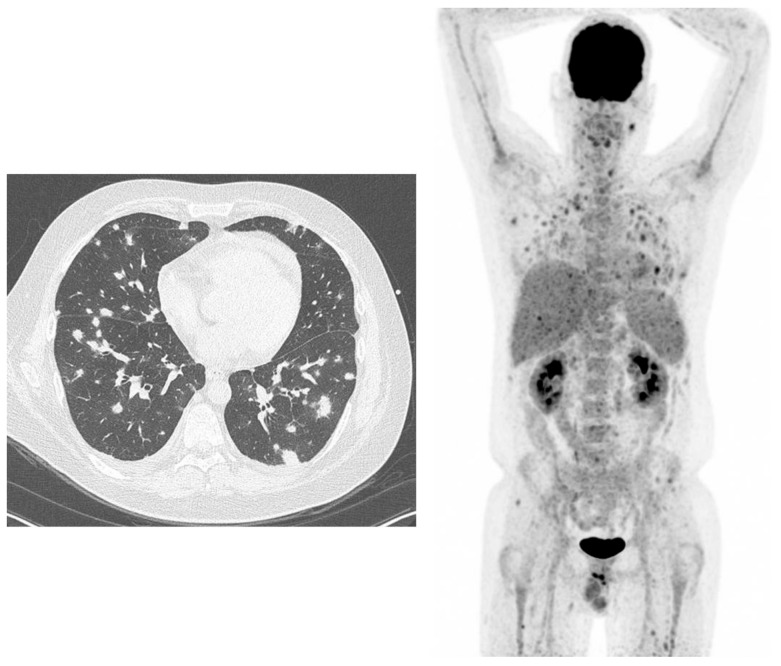
38-year-old male, known with acute lymphatic leukaemia. Because of fever and bacteraemia he was treated with meropenem (antibiotics). High-resolution computed tomography (HR-CT) (left figure): several lung densities with irregular borders, not typical for bacterial infections, but typical for angio-invasive aspergillosis, because of the halo sign. ^18^F-fluorodeoxyglucose positron emission tomography/computed tomography (^18^F-FDG-PET) (right figure): not only lung involvement but also infectious lesions in the liver, spleen and in muscles throughout the body.

For staging patients with IFIs, the overall agreement of published studies is that ^18^F-FDG-PET is useful. It detects pathophysiological changes before anatomical imaging techniques, it is able to image infectious foci and extent also outside the lungs (Figure 1), and it may pick up infectious foci which are not yet clinically apparent. However, as ^18^F-FDG-PET is an aspecific imaging technique, there is always the need for confirmation by biopsy. ^18^F-FDG-PET is able to define sites of most active infection suitable for biopsy [42].

**Figure 2:**
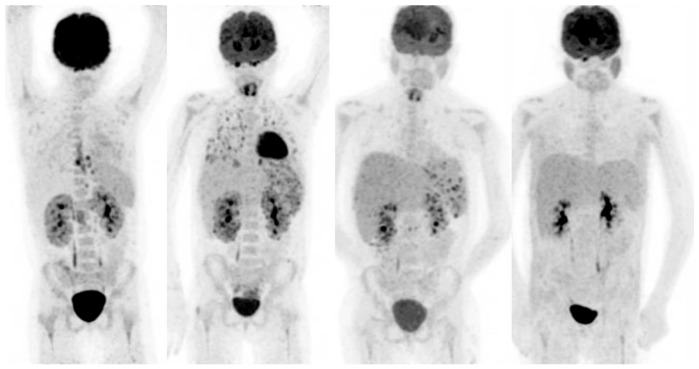
20-year-old female, known with recurrent acute lymphatic leukaemia. After first chemotherapy cycle fever and neutropenia. Blood cultures: candidia. Left scan: infectious foci around catheter in the oesophagus and heterogeneous uptake in the kidneys. Scan 2, 6 weeks after treatment with fluconazole: progressive disease with now involvement of the lungs, spleen and kidneys. Biopsy of the parenchyma of the right kidney confirmed renal candidiasis. Caspofungin was added to the antifungal treatment. Scan 3, 3 months after treatment with fluconazole and caspofungin: improvement, but still involvement of spleen and kidneys. Treatment continued. Scan 4, after 6 months treatment: no active infection anymore.

For therapy evaluation, the role of ^18^F-FDG-PET is much larger and, if confirmed in large prospective studies, can be considered the first follow-up imaging modality of choice. Patients with IFIs often require antifungal drug treatment for a long time period (even up to years) and therapy is very expensive. Lesions on CT and MRI may persist for a long time even after successful treatment due to e.g., fibrosis. There is an absolute need for an imaging test that is able to tell the clinician: (1) there is no active infection anymore, you can stop the treatment, (2) there is progression of infection, you have to modify the treatment, or (3) therapy is adequate, but the infection is not solved yet, you should continue the treatment. Till now, mainly case reports exist in literature, but all agree that ^18^F-FDG-PET has added value: it can serve as a valuable tool for monitoring treatment response (Figure 2), leads to change in therapy in cases of poor response, may help to determine when antifungal agents do not effectively reach the site and surgery should be recommended, is helpful to stop therapy in cases with inactive disease at a time point when lesions on other imaging modalities have not completely resolved, and helped in deciding the best time point (no active infection anymore) for autologous stem cell transplantation in patients with leukaemia/lymphoma [43].

Very recently, three retrospective single-centre studies confirmed the utility of ^18^F-FDG-PET/CT to detect IFI in immunocompromised patients and, secondly, to evaluate the follow-up by monitoring the treatment response, thereby helping to identify when patients are responders, and to assist in guiding the appropriate duration of therapy [44,45,46].

To our opinion, ^18^F-FDG-PET serves as a valuable imaging tool that is able to raise suspicion of an IFI (but culture or biopsy still required), to detect all fungal lesions within a patient, and offers a unique possibility in monitoring therapy efficacy.

## 4. Radiolabelled Somatostatin Analogues in Rheumatoid Arthritis

Molecular imaging techniques are promising tools to support in the early diagnosis and monitoring of therapy in various rheumatic diseases. Nowadays, there is an increasing emphasis on diagnosing inflammatory diseases in the preclinical stages in order to promptly start an adequate treatment ideally preventing irreversible tissue damage. Since the diagnosis of rheumatic disease and the assessment of disease activity are moving towards the subclinical end of the disease spectrum, nuclear molecular imaging techniques may become of increasing importance [47]. Target probes for RA able to interact with specific targets have been described, making it possible to image biological processes at the cellular or molecular level [48] and various nuclear imaging techniques are available for this purpose [49]. Reubi et al. identified overexpression of somatostatin receptors in active RA synovia [50]. These findings were confirmed by others and have served as nourishment not only for therapy but also for diagnostic purposes [51,52,53,54,55]. For SPECT, many radiolabelled somatostatin analogues with ^111^In and ^99m^Tc are available, such as ^111^In-octreotide, ^99m^Tc-depreotide and ^99m^Tc-HYNIC-TOC with different affinities for receptors 2, 3 and 5. (^99m^Tc)-HYNIC-TOC allows to visualize the presence of inflammation in the synovial tissue thus it could be useful in order to select patients that could benefit of unlabelled somatostatin for therapy. Anzola et al., in a pilot study of eighteen patients with RA not responding to conventional treatment, demonstrated intense uptake in all ^99m^Tc-HYNIC-TOC scans with a symmetric uptake pattern in hands, and, to a lesser frequent, in knees, shoulders and ankles in agreement with clinical manifestations [56]. Eleven out of the 18 patients were re-evaluated with a new scan after an anti- TNFα treatment (infliximab) and a statistically significant decrease in tracer uptake was found compared to the baseline scan. This finding was in agreement with the theory that somatostatin receptors can be overexpressed in active phases of the disease characterized by endothelial activation and lymphocyte infiltration of the synovial cells [50]. All patients with positive findings at the baseline ^99m^Tc-HYNIC-TOC scan, showed a clinical improvement after infliximab therapy. This pilot study suggested how this molecule is able to identify patients with active disease and likely to respond to anti TNFα therapy. In a series of 14 patients with RA, Vanhagen et al. reported that 76% of the swollen joints were visualized by ^111^In-octreotide and the degree of pain and swelling correlated well with positive findings in the joints [15].

RA can also be successfully imaged by ^18^F-FDG-PET [57]; however, although it was found to be highly sensitive in identifying synovial inflammation, this technique is not able to identify the pathophysiological event involved [58]. PET tracers that specifically bind to somatostatin receptors (^68^Ga-DOTA peptides) have been developed and are used to diagnose patients with neuroendocrine tumours [59]. Among the different tracers, ^68^Ga-DOTA-NOC has the broadest somatostatin receptors subtype affinity [60] and a favourable dosimetry. In consideration of the increased expression of somatostatin receptors on fibroblasts and on inflammatory cells, those ^68^Ga-DOTA tracers could potentially also be used for the identification of inflammatory process in patients with RA [61] offering the advantages of a better images quality and anatomic definition compared with ^99m^Tc-HYNIC-TOC scan.

## 5. Radiolabelled Anti-CD20 and Anti-Tumor Necrosis Factor (Anti-TNFα) Monoclonal Antibodies in Rheumatoid Arthritis

Besides the aforementioned radiopharmaceuticals, other new imaging techniques might also help the referring clinician in therapy decision-making and follow-up [62]. In particular, recent studies evaluated the possible application of scintigraphy with ^99m^Tc-anti-TNF MoAb in the assessment of patients affected by IBD and inflammatory arthropathies, such as RA, treated by intra-articular infliximab [63,64]. Biological agents directed against TNF changed the therapeutic approach of these patients, due to the innovative mechanism of action, and based on the ability to recognize specific molecular or cellular targets involved in the disease pathogenesis. The administration of anti-TNF drugs led to a significant improvement in the prognosis of patients refractory or intolerant to conventional disease-modifying anti-rheumatic drugs (DMARDs) treatment. Randomized controlled trials demonstrated the efficacy of these drugs to prevent the development of bone erosive damage, with consequent better profile in terms of remission status and prevention of long-term disability [65]. Indeed, TNF is a pro-inflammatory cytokine able to promote synovial inflammation and bone and cartilage erosions [66]. Produced by several different immune and non-immune cell types, it was recognized as a key factor in the inflammatory process of arthropathies, such as RA, psoriatic arthritis (PsA) and ankylosing spondylitis (AS) [67]. To date, five different anti-TNF drugs have been developed: infliximab, adalimumab, golimumab, certolizumab pegol, which are MoAbs or fragments, and etanercept, a genetically engineered fusion protein composed of a dimer of the extracellular portions of human TNFR2 fused to the Fc portion of a human IgG1 [67]. Despite the recognized efficacy of anti-TNF agents, approximately one third of patients has to discontinue the treatment due to inefficacy or intolerance. In these cases, the switch to another anti-TNF agent could represent a valid option due to significant differences in terms of molecular structure, pharmacokinetics, interactions with TNF, generation of antibodies, induction of apoptosis, and dosing regimen among the TNF antagonists [65]. More recently, other molecular or cellular targets involved in the pathogenesis of the autoimmune diseases have been identified, such as the pro-inflammatory cytokines IL1 and IL6, Cytotoxic T-Lymphocyte Antigen 4 (CTLA-4), and molecules involved in the activation, differentiation and maturation of B lymphocytes. In particular, recent studies demonstrated the involvement of B lymphocytes in RA and the benefits of rituximab treatment in patients. These drugs have been used in patients that do not benefit from anti-TNF drugs, obtaining good results in terms of remission and long-term prognosis [65].

### 5.1. (^99m^Tc)-Infliximab Scintigraphy

**Figure 3:**
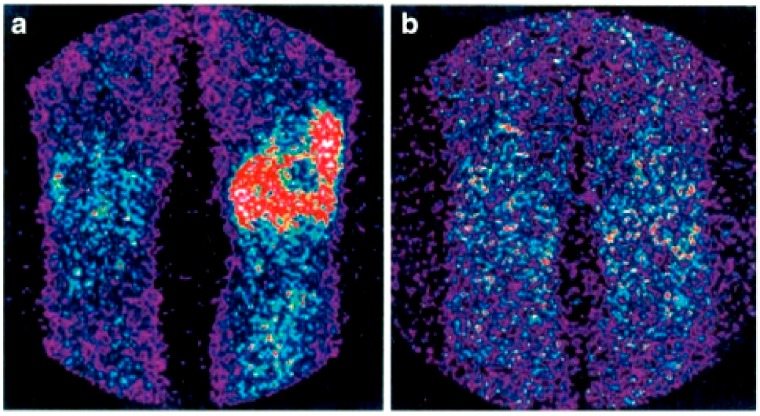
Scintigraphy with ^99m^Tc-infliximab before (**a**) and 4 months after (**b**) intra-articular administration of infliximab. Red colour represents uptake of the ^99m^Tc-infliximab in scintigraphy (from Conti, F.; et al. *Arthritis Rheum,* **2005**, *52*, 1224–1226 [68]).

The chimeric mouse/human antibody infliximab demonstrated a good efficacy in the treatment of patients with inflammatory arthropathies since it can bind both soluble and membrane-bound TNF with high affinity and specificity. Therefore, the use of the same antibody radiolabelled with ^99m^Tc was explored by Conti et al., with promising results. In this case report, they demonstrated the possibility to use ^99m^Tc-infliximab scintigraphy to evaluate the articular presence of TNFα, thus monitoring the response to anti-TNFα therapy [68]. The radiopharmaceutical was injected in a patient affected by spondylarthropathy (SpA) with a mono-arthritis resistant to conventional intra-articular and systemic treatment. After intravenous injection of the radiopharmaceutical, planar images of the involved joints were acquired at 6 h and 24 h. As shown in Figure 3, high uptake of radiolabelled infliximab was observed in the right knee revealing the presence of TNFα. Therefore, the patient was treated by intra-articular infliximab (100 mg) and underwent a control scintigraphy at the end of the treatment showing the disappearance of TNFα from the inflamed joint. Remission was also confirmed by clinical and ultrasonography assessment. A larger population was then evaluated including 12 patients with active refractory mono-arthritis (11 knees and 1 ankle), affected by RA (5 patients) and SpA (7 patients) [69]. At baseline and after 12 weeks of treatment with intra-articular infliximab (100 mg for the knee and 50 mg for the ankle), a ^99m^Tc-infliximab scintigraphy was performed. Quantitative analysis of TNFα uptake in the inflamed sites was performed by calculating the target-to-background (T/B) ratios [69]. The results of this study confirmed the promising role of ^99m^Tc-infliximab scintigraphy in monitoring the response to anti-TNFα therapies. Indeed, a significant decrease in the T/B ratio of the affected joints was associated with complete remission as a confirmation of the success of the treatment. Overall, responder patients showed a significantly higher increase in uptake from 6 h to 20 h compared to non-responders. Therefore, the high level of TNF confirmed by ^99m^Tc-infliximab scintigraphy in the inflamed joints is associated with better efficacy of intra-articular administration of infliximab. The same technique was then compared to MRI and clinical examinations in 8 patients for a total of 198 joints [70]. Scintigraphy correlated with clinical examinations and showed high sensitivity and specificity (89.8% and 97.3%). MRI showed both lower correlation coefficients and sensitivity and specificity (59.2% and 65.3%).

### 5.2. ^99m^Tc Radiolabelled Adalimumab Scintigraphy

**Figure 4:**
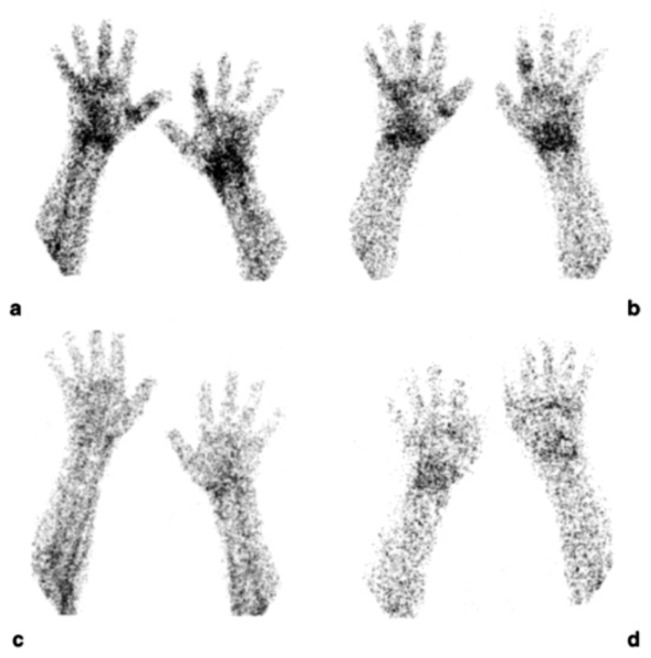
Scintigraphic images of wrists of a rheumatoid arthritis (RA) patient injected with (^99m^Tc)-adalimumab (anti-TNFα mAb) before (**a** and **b**; dorsal images after 6 and 20 h p.i., respectively) and 3 months after systemic therapy with adalimumab (**c** and **d**; dorsal images after 6 h and 20 h p.i., respectively), (from Malviya, G.; et al. *Q. J. Nucl. Med. Mol. Imaging* **2008**, *52*, 13–14) [71].

Adalimumab was the first “fully humanized” anti-TNFα antibody approved by the Food and Drug Administration (FDA). Like infliximab, it was successfully radiolabelled with ^99m^Tc using the same method and tested in patients affected by RA [63]. Each patient received a sub-therapeutic dose of 0.1 mg (740 MBq) and whole-body and joint-specific images were acquired at 5 min, 4 h, and 24 h post-injection. Uptake was observed in inflamed joints after 4 and 24 h (median increased uptake was 30%) but some joints did not accumulate the radiopharmaceutical since they did not express TNFα. Malviya et al., compared in a pilot study the use of ^99m^Tc-adalimumab and ^99m^Tc-infliximab scintigraphy in 12 and 9 patients, respectively, affected by RA [71]. Scans were performed before and 3 months after intra-articular therapy with infliximab or systemic therapy with adalimumab. Planar anterior and posterior images were acquired at 6 and 20 h after injection of ^99m^Tc-infliximab or ^99m^Tc-adalimumab (370 MBq). Region of interests (ROIs) were drawn and T/B ratio was calculated in all affected joints (Figure 4). There were no differences in the biodistribution of tested radiopharmaceuticals. However, the degree of joint uptake was variable and, in some cases, did not correlate with pain or swelling. After therapy, reduction of clinical symptoms correlated with reduced uptake of both radiopharmaceuticals in affected joints. Therefore, it was concluded that ^99m^Tc-TNFα antibodies could be used for therapy decision-making in patients with active RA being predictive of success of therapy with same unlabelled MoAb.

### 5.3. ^99m^Tc-Rituximab Scintigraphy

^99m^Tc-rituximab, a chimeric murine/human MoAb that specifically targets CD20 expressed by B lymphocytes, was developed to image infiltration of these cells in autoimmune diseases or in non-Hodgkin’s lymphomas (NHLs). The CD20 antigen is not expressed by other hematopoietic cells, therefore ^99m^Tc-rituximab offers the possibility to specifically image transformed B lymphocytes for diagnosis and for programming a tailored therapy. The utility of anti-CD20 imaging in RA has been demonstrated by Malviya et al., in five patients revealing a mild to moderate uptake in affected joints [18]. A similar study was performed using ^124^I-rituximab for PET imaging in five other patients affected by RA. Images were acquired up to 96 h after injection to allow clearance of the radiopharmaceuticals from circulation, thus trying to increase the T/B ratio. Also in this study, rituximab scintigraphy was shown to be feasible and useful to evaluate B cell infiltration in affected joints but, at the moment, the usefulness of ^124^I-rituximab in the diagnosis and follow-up of RA is not justified by the high costs [72].

The positivity at anti-CD20 scintigraphy could be the basis for the start of a treatment with cold antibody. Relapsing or refractory NHL represent a paradigmatic example of the possibility to perform therapy decision making with NM imaging. Ibritumomab is an anti-CD MoAb similar to rituximab that is commercially available in Europe for the treatment of NHL alone or in association with a β−emitting radioisotope, mainly with ^90^Y (Zevalin) or ^131^I or ^177^Lu, for radio-immuno-therapy. A pre-therapy scintigraphy using ^111^In is generally performed in order to assess CD20 expression an to predict the subsequent the Zevalin biodistribution. The same approach could also be applied for autoimmune disorders.

### 5.4. ^99m^Tc Radiolabelled Certolizumab Pegol Scintigraphy

Certolizumab pegol is an engineered humanized monoclonal antibody Fc-free Fab′ fragment specific for human TNFα. This antibody was indirectly labelled with ^99m^Tc using HYNIC as a bi-functional chelator. As with the aforementioned radiopharmaceuticals, ^99m^Tc-certolizumab pegol scintigraphy was performed in normal subjects and 5 patients with RA [73,74]. Imaging was performed before and 12 and 24 weeks after therapy with the cold antibody. All patients also underwent MRI and US imaging and authors concluded that they were able to demonstrate a significant predictive value of the immunoscintigraphy at the joint level.

Concluding, In the light of the possibility to choose among different biological drugs, with specific targets, the establishment of a personalized treatment is a critical issue. Several efforts have been performed in order to identify biomarkers, in particular different kind of MoAbs, able to predict the response to treatment and guide the clinician in the therapy decision-making.

## 6. ^18^F-FDG-PET/CT to Image Large Vessel Vasculitis

Systemic vasculitis is an inflammatory blood vessel disorder which, if untreated, may lead to arterial obstruction, or to the rising of aneurysms with consequent vascular damage and rupture. Therefore, there is a need to recognize them as early as possible. Typically, vasculitis are classified according to the calibre and type of vessel predominantly involved [75]. In particular, several classifications of vasculitis are currently available (American College of Rheumatology criteria, the Chapel Hill Consensus Conference definitions, the adapted Zeek classification system), but the low specificity of inflammatory laboratory parameters and the frequently atypical presentation of these diseases, make the diagnosis a great challenge. Nuclear medicine has made a “giant leap” in terms of diagnostic performance in systemic vasculitis, thanks to the development of “hybrid” PET/CT camera systems. In recent years, ^18^F-FDG-PET/CT has been the most explored imaging technique for the diagnosis of large vessel vasculitis (LVV), which consists of giant cell arteritis (GCA) and Takayasu’s arteritis (TA) and its use is recommended by recently published guidelines [76]. Pathophysiology of LVV is represented by a vivid inflammatory cascade with T-cell and activated macrophage recruitment and accumulation into the vessel wall. An overexpression of glucose transporters (GLUT-1, GLUT-3), and an increased hexokinase type II (HK-II) activity, stimulated by cytokines or mutagens, lead to an incremental glucose consumption, and a consequent increased ^18^F-FDG uptake by inflammatory cells entrapped in the vessel wall [77]. Inflammatory cell infiltration revealed by ^18^F-FDG-PET/CT anticipates the development of oedema of the vessel wall depicted by morphological imaging, giving the possibility to diagnose LVV in an early stage of disease [78]. Diagnosis, evaluation of the extent of the disease, and monitoring the response to treatment are the most common indications for the application of ^18^F-FDG-PET/CT in LVV [79].

### 6.1. Diagnosis, Extent and Impact of (^18^F)-FDG-PET/CT on the Management of Large Vessel Vasculitis (LVV)

On the basis of the available scientific literature, ^18^F-FDG-PET/CT shows a pooled sensitivity and specificity of 89% and 98% in GCA, and of 84% for both sensitivity and specificity in TA, although a very recent meta-analysis reported a pooled sensitivity and specificity equal to 81% and 74%, respectively, for TA activity [80,81]. Utility in the evaluation of extra-cranial involvement of disease in patients affected by temporal arteritis (whole-body scan), and in the identification of areas of increased ^18^F-FDG uptake in which a biopsy should best be taken to obtain a definite diagnosis, are some additional advantages of ^18^F-FDG-PET/CT [82]. On the other hand, false positive results, mainly due to observed increased ^18^F-FDG uptake in atherosclerotic vessels (likely to be related to macrophage-rich areas in atherosclerotic plaques), and low spatial resolution of PET and PET/CT scanners currently employed, are possible limitations [83]. One of the major difficulties in using ^18^F-FDG-PET/CT for the assessment (diagnosis and response to treatment) of LVV is the heterogeneity in the evaluation systems used by nuclear medicine physicians [84]. Visual assessment is the most commonly used grading system currently. Meller et al. in 2003 proposed a four-point grading scale, in order to graduate inflammatory intensity of the vessel wall, comparing uptake in the large vessels to hepatic ^18^F-FDG uptake (“0” = no uptake; “1” = uptake inferior to the liver; “2” = uptake similar to the liver; “3” = uptake superior to the liver) [85]. High specificity (about 100%), but variable sensitivity levels between 56% and 77%, and a high inter- and intra-observer reproducibility (90% and 93% respectively) were observed [86]. Uptake pattern (linear, segmental, or focal) and intensity of the vessel wall uptake, also must be taken into consideration. Generally, in LVV the uptake pattern of ^18^F-FDG is "homogeneous" (or initially segmental), while a "focal or heterogeneous" uptake pattern can easily be attributable to atherosclerotic plaques (recognizable at the CT component of PET). A recent published study showed that by up to 45% of control patients without LVV ^18^F-FDG uptake can be seen with a vascular uptake grade 1 [84]. Furthermore, in 14% grade 2 can be observed, and in 4% grade 3 can be observed. More recently, a principal component analysis (PCA) and cluster analysis (CA) were performed with the aim to compare the differential vascular ^18^F-FDG uptake patterns in TA and GCA patients. Results showed similar arterial uptake patterns between TA and GCA patients, in particular symmetric in paired vessels and contiguous in the aorta, and minimal differences in the aorta segments, suggesting TA and GCA as “part of the same disease spectrum” and not distinct conditions [87]. The same group evaluated the aortic dilatation in patients with LVV by the use of ^18^F-FDG in a longitudinal study. They reported an increased aorta diameter in LVV patients than controls comparing the first and last PET/CT (median interval time of 31 months) in association to significant predictors factors of aortic dilatation such as male sex and, only for GCA, hypertension [88].

According to a large consensus, a vascular uptake degree superior to the liver (grade 3) is highly specific for LVV, while if the uptake is lower than the liver (grade 1) presence of LVV can be likely excluded [83]. The interpretation of grade 2 (vascular uptake similar to liver) is still unclear and we may consider it as an intermediate probability of LVV. In a recent study, the strong correlation between PET scores and inflammation markers (erythrocyte sedimentation rate and C-reactive protein), particularly when using vessel to liver ratios, was higher than CT scores alone in patients with known LVV, confirming the aforementioned findings [89].

Semi-quantitative methods (SUV_max_, total vascular score, arterial-to-reference organ ratio) may be used in the case of a doubtful diagnosis at visual assessment. Because there is a significant overlap of SUV_max_ between controls and LVV patients, the specificity for the use of SUV_max_ was low, and since there is no clear threshold its use is still debated [90]. Calculation of the total vascular score (TVS) is another controversial approach, and still not shared. TVS takes into account seven vascular regions (carotid arteries, subclavian, axillary, iliac, femoral, thoracic aorta, abdominal aorta), by assigning an uptake score (from “0” to ”3”) for each region, based on the degree of intensity, leading to a maximum score of 21. A TVS equal and/or superior to 6 ± 0.2 is considered highly specific for the presence of vascular disease [91]. Calculation of the ratio between SUV_max_ in the vessel and SUV_max_ of a reference organ is an approach based on vascular and parenchymal SUV_max_ values of reference tissue (liver, lung, vascular pool) and, therefore, reflects the limits already indicated above. At present, there is no absolute consensus of what is the best reference organ to use.

^18^F-FDG-PET/CT has a significant impact on the management of patients with suspected LVV, especially when performed in patients not receiving immunosuppressive treatment. Fuchs et al. reported changes in the treatment recommendation in 26.7% of patients not receiving immunosuppressive medication and in 22.6% of patients receiving immunosuppressive drugs. The diagnostic accuracy decreased after the institution of immunosuppressive therapy (93.3% vs. 64.5%) [92].

Recently, in order to have better information about vessel wall changes reducing the exposition to radiation (particularly important in young patient), fully integrated PET/MRI systems have been evaluated in LVV. A pilot study demonstrated that ^18^F-FDG PET/MRI is feasible in LVV with visual and semi-quantitative results highly comparable to PET/CT. Furthermore, PET/MRI holds promise for a precise determination of the disease extent and disease activity in LVV [93].

### 6.2. Role of (^18^F)-FDG-PET/CT in Monitoring the Treatment Response in LVV

**Figure 5:**
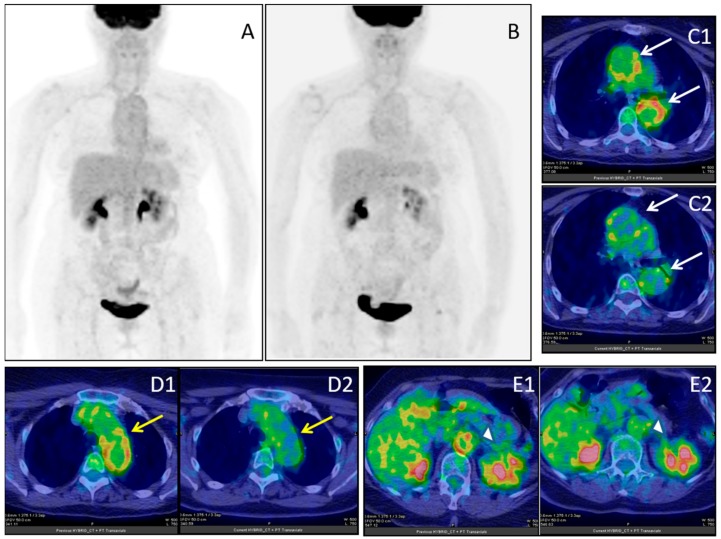
Female, 64 years-old; (^18^F)-FDG-PET/CT MIP in giant cell arteritis (GCA) before (**A**) and after (**B**) immuno-suppressive treatment; ascending and descending aorta (white arrows), before (**C1**) and after (**C2**) treatment; aortic arc (yellow arrow), before (**D1**) and after (**D2**) treatment; abdominal aorta (white arrow-head), before (**E1**) and after (**E2**) treatment.

Changes in vessel activity during disease and after treatment reduce the diagnostic accuracy but create a further application of ^18^F-FDG-PET/CT, namely the evaluation of the response to treatment (Figure 5). In recent studies, SUV_max_ reduction can be notable three months after the initiation of the treatment, while no more reduction between three and six months after the end of the therapy was found [82,83]. In our experience, most responders to treatment still maintain a grade 1–2 visual vascular uptake. This is probably due to: (1) minimal persistent inflammation of the vessel wall after treatment, (2) vascular remodelling (because vascular smooth muscle cells also take up ^18^F-FDG), and (3) resistance to treatment (similar to what happens for antibiotics).

Meller et al. suggested that ^18^F-FDG-PET may be more reliable than MRI in monitoring disease activity during immunosuppressive therapy [85]. Also, several groups demonstrated that ^18^F-FDG-PET/CT is a reliable and accurate tool for monitoring therapy in conjunction with clinical and biochemical findings, also in patients with poor clinical therapy response or suspected relapse [94,95,96]. Furthermore, in TA, both MRI and ^18^F-FDG-PET were found to be promising for the assessment of disease activity [97]. A clear response can be documented by ^18^F-FDG-PET many times if a baseline scan is available [98].

LVV is an often-reported cause of fever of unknown origin in elderly people. Patients with LVV have significantly higher aortic wall scores by ^18^F-FDG-PET/CT, and aortic wall thicknesses by contrast-enhanced CT compared to controls. A significant improvement in aortic wall thickening can be witnessed by a decrease in PET scores and by contrast-enhanced CT findings in patients after treatment [99].

Since ESR or CRP alone is not considered to be a reliable parameter to assess disease activity in large-vessel vasculitis, many authors include ^18^F-FDG-PET/CT as an imaging modality in the evaluation of new treatments [100,101].

In the near future, the concomitant development of increasingly powerful PET/CT scanners, the development of new radiopharmaceuticals (more specific for inflammation), and the increased use of PET/MRI hybrid scanners probably will lead to a next step forward in the diagnosis and clinical management of LVV, despite the lack of a “gold standard” for evaluating disease activity of LVV [81]. Indeed, very recently, ^18^F-FDG-PET/CT and 3D-black-blood 3T-MRI were compared for the diagnosis of LVV, considering them both useful in the LVV diagnosis with high diagnostic accuracies [102].

## 7. Imaging Immunological Network in Cancer

It is well established that the tumour microenvironment (TM) plays a pivotal role in driving or slowing down the cancer progression due to the coexistence of stromal cells, malignant cells, immune cells, and/or endothelial cells that contribute to the growth of the tumour. Indeed, in the TM several subsets of leukocytes are present and, depending on their polarization status, these may have a pro- or anti-tumoral function through the secretion of biochemical signals such as cytokines, chemokines, or growth factors. Therefore, a better comprehension of the interplay between TM components and biochemical products, and their complex interaction with the host immune system, would provide new biological targets for nuclear medicine imaging and treatment monitoring [103].

Surgery, chemotherapy, radiotherapy and targeted drugs are the “normal” treatment options in patients with metastasized cancer. Just recently, immunotherapy was added as the fifth weapon in the arsenal of the treating clinician. Cancer immunotherapy was in 2013 selected as “Breakthrough of the Year” by Science, as the editors concluded that long-sought efforts to unleash the immune system against tumours are paying off, but they also stated that researchers are largely clueless as to which patients do benefit and that biomarkers have to be identified to offer answers and to experiment with ways to make therapies more potent [104]. The numbers of available immunotherapeutic drugs are rapidly increasing and several monoclonal antibodies are already approved by the FDA. As stated in the *Science* editorial, selection of patients in which the expensive immunotherapy will help is difficult; furthermore, severe toxicity of the immunotherapeutic drugs is a serious problem, resulting in so-called immune-related adverse events (irAEs) due to the increased reactivity of T cells [105].

The immune system is regulated by a complex system including multiple checkpoints that control the homeostasis. T lymphocytes are the cells of the immune system that identify and destroy tumour cells. However, cancer cells use these checkpoints to escape detection, e.g., by camouflaging themselves with a shield of molecules called programmed cell death protein ligand 1 (PD-L1). Lymphocytes possess PD-1 receptors, bind to the PD-L1 on the tumour cells, thereby destroying their capacity to attack. The new immunotherapeutic drugs are antibodies that block PD-1 from the immune system cells or PD-L1 from the tumour cells, leading to lymphocytes to regain their defence potential. The PD-1/PD-L1 checkpoint and the CTLA4 checkpoint are the most extensively studied targets for more efficacious and precise cancer immunotherapy, especially in non-small-cell lung cancer (NSCLC) [106,107]. The expression of PD-L1 by TM can represent a biomarker to predict the response to immunotherapy: tumours that show low expression of PD-L1 will not respond to these drugs. Therefore, the use of a radiolabelled anti-PD-L1 probe could provide non-invasive information about a tumour’s PD-L1 expression, avoiding biopsies, and could be useful in order to select patients candidates to immunotherapy and to predict the response to treatment [108,109].

Another molecule that is nowadays largely used to study the tumour environment is interleukin-2 (IL2). It can be used as a surrogate marker to image activated T lymphocytes and regulatory T-cells and it can be radiolabelled for both SPECT and PET imaging. IL2 has been extensively studied in several tumours, including melanoma [110], squamous cell carcinomas of head and neck [111], and renal cell carcinoma [112], showing optimal biodistribution, dosimetry and high T/B ratio.

Molecular imaging is able to provide information about the expression levels of targets and receptors, and may play a role in predicting which patients may benefit from immunotherapy and may also help in early therapy evaluation, thereby avoiding severe side effects and unnecessary therapy [113].

At this moment, for disease monitoring during immunotherapy, ^18^F-FDG-PET is still mainly used, although we know that this has several limitations.

### 7.1. Programmed Cell Death Protein (PD-1) Imaging

Currently, two FDA-approved antibodies that target PD-1 are available: pembrolizumab and nivolumab, both used since a couple of years in metastatic melanoma and metastatic lung cancer, but indications are extending. ^64^Cu-DOTA-anti-PD-1, a murine monoclonal antibody, was tested in a mouse model of melanoma and accumulated effectively in the tumour. Blocking studies were performed to validate the specificity of the tracer for the tumour [114]. These findings were confirmed by another group with a slightly different ^64^Cu labelled anti-PD-1 tracer [115]. Just recently an anti-PD-1 human antibody was labelled with both ^89^Zr and with ^64^Cu to image immune cell status in mice bearing human skin melanoma tumours showing a more favourable biodistribution and higher tumour to muscle ratios with the ^89^Zr variant [116]. Positive results of ^89^Zr-nivolumab were recently confirmed in a humanized murine model of lung cancer and, secondly, in healthy non-human primates to test a preliminary biodistribution and clearance [117,118].

^89^Zr-pembrolizumab was also evaluated in murine models, and allowed direct visualization of human peripheral blood mononuclear cells engrafted in mice, demonstrating to predict human dosimetry too [119,120]. The first results of clinical studies with ^89^Zr-pembrolizumab or ^89^Zr-nivolumab are expected soon (clinicaltrials.gov, NCT02760225).

### 7.2. Programmed Cell Death Protein Ligand 1 (PD-L1) Imaging

Atezolizumab is an antibody targeting PD-L1 and is FDA approved for metastatic lung cancer, advanced bladder cancer and urothelial carcinoma. Several preclinical studies showed specific accumulation of radiolabelled (with ^111^In for SPECT imaging, with ^64^Cu and ^89^Zr for PET imaging) PD-L1 targeting antibodies in PD-L1 positive tumours. Furthermore, high and low PD-L1 expression could be noticed, as well as physiological uptake in lymphoid tissue and organs such as spleen, thymus, lymph nodes and brown fat [115,121,122,123,124,125,126]. Luckily, this approach was also already tested clinically. ^89^Zr-atezolizumab was tested in patients with metastatic lung cancer, bladder cancer or triple-negative breast cancer, and showed high uptake in tumour lesions with large heterogeneity between lesions, patients and tumour types, but also showing a promising role for the assessment of clinical response prediction (clinicaltrials.gov, NCT02453984 and NCT02478099) [127,128]. Recently, new compounds, including affibody, small high-affinity engineered protein and recombinant human antibody were tested for PD-L1 imaging and monitoring treatment response in preclinical tumour models. These molecules were radiolabelled with different PET isotopes and obtained a clear visualization of PD-L1 positive tumours in comparison to PD-L1 negatives, suggesting them as new radiopharmaceuticals with translational applications for human immune-checkpoints imaging [129,130,131].

Unfortunately, at the moment, the possible role of radiolabelled anti PD-L1 for both diagnostic and therapy decision making purposes is only theoretical and many aspects need to be further addressed, but we expect in next future that this molecule could gain a role in the management of several oncologic diseases. Moreover, it will be desirable to conjugate anti-PD-L1 with an isotope suitable for radio-immunotherapy in order to increase the therapeutic efficacy of the cold molecule.

### 7.3. CTLA-4 Imaging

CTLA-4 targeted antibodies have shown efficacy in the treatment of cancers; it provided therapeutic benefits, but can also lead to severe adverse effects. Clinical trials were performed with ipilimumab and tremelimumab, showing tumour response rates as mono-therapy in approximately 10%. So, these therapeutic drugs have been shown extremely effective but only in a subpopulation of patients; molecular imaging could assist in selecting those patients that are more likely to respond to anti-CTLA-4 immunotherapy [127]. A ^64^Cu-labelled anti-mouse CTLA-4 antibody was developed and visualized CTLA-4 positive colon tumours in mice [132]. This radiopharmaceutical, however, has to be evaluated in models with varying levels of CTLA-4 expression to eventually be used in patient studies. Recently, a PEGylated single-domain antibody fragments (VHHs) was labelled with ^89^Zr in tumoral preclinical models in order to evaluate the CD8^+^ T cells and the treatment response to anti-CTLA-4 immunotherapy. Experimental data showed not only the ability of ^89^Zr-PEGylated-VHH-X118 to provide the precise distribution of T cells within the tumour, but also to predict the anti-CTLA-4 immunotherapy response in relation to T cells signal, if homogeneously distributed (responders) or heterogeneously (not responders) [133].

### 7.4. CD25 as Target for Imaging Tumour-Infiltrating Lymphocytes

Another approach to image the immune system is by targeting lymphocytes involved in the immune process. Best results so far have been achieved by using radiolabelled IL2 to target CD25, IL2 receptors (IL2R), expressed on activated T lymphocytes and T-regulatory cells. IL2 is a cytokine involved in many pathological conditions characterized by a chronic lymphocytic infiltration. Studies using radiolabelled IL2 started in the 1980s and first radioisotopes used were ^125^I and ^35^S for in vitro and animal studies [134,135,136]. Radiolabelled IL2 was extensively studied in animal models and patients with diabetes [137,138,139,140], in order to research T lymphocyte infiltrates in rats’ renal allografts [141], in Crohn’s and coeliac disease patients [142,143], in other autoimmune disorders [144,145,146] and for imaging of vulnerable atherosclerotic plaques [147,148].

Chianelli et al. developed a new method to radiolabel IL2 with ^99m^Tc and this radiopharmaceutical was intensively studied in many of the pathological conditions, providing in vivo measurements of IL2R positive cells and, therefore, valuable prognostic information for the selection of patients who may benefit from IL2 immunotherapy [145]. In the following years, a new labelling procedure for IL2 with the positron emitting radioisotope ^18^F was developed to allow PET imaging of the IL2R [149]. IL2 has been recently labelled with ^18^F by conjugation with succinimidyl 4-^18^F-fluorobenzoate (^18^F-SFB) for in vivo imaging of tumour-infiltrating CD25^+^ activated T lymphocytes and for CXCR4 antagonist therapy monitoring, obtaining promising results that suggest ^18^F-FB-IL2 as a clinical non-invasive tool for cancer imaging and driving immunotherapy choice [150]. More recently, a mutant of IL2 was developed and radiolabelled with ^18^F with the aim of investigating its pharmacokinetic and binding properties compared to wild-type IL2. Results showed a lower binding affinity for IL2R and slower kinetics of mutant IL2 that could be advantageous for therapeutic use, although further studies are required [151].

At present, although not yet commercially available, radiolabelled IL2 is available for diagnostic purposes and for therapy decision making.

As far as the imaging of T lymphocytes in the tumour environment is concerned, both iodinated and techenetiated IL2 have been used for imaging of squamous cell carcinoma of the head and neck [111], renal cell carcinoma [112] and in melanoma [110]. In particular, in a population of 30 patients with primary cutaneous melanoma and studied with ^99m^Tc-IL2, CD25 was detected in TILs in 15 tumours of which 11 demonstrated ^99m^Tc-IL2 uptake by imaging. In 3 of the 4 tumours considered negative by ^99m^Tc-IL2 scintigraphy, the level of CD25^+^ TIL was less than 15% suggesting that the low number of TIL may have been insufficient for discrimination of the T/B ratio by ^99m^Tc-IL2 imaging. Therefore, ^99m^Tc-IL2 is a reliable tool to estimate the entity of TIL at histological analysis thus hypothetically allowing a selection of patients that will respond to immunotherapy. Indeed, a patient with a high number of TILs is expected to show a good response to immune checkpoint inhibitors whereas a patient with a low number of TILs could have a bad response and bad prognosis.

In a recent phase I preliminary safety study on 5 patients with metastatic melanoma, ^99m^Tc-IL2 scintigraphy was tested in patients undergoing immunotherapy either with ipilimumab or pembrolizumab. ^99m^Tc-IL2 showed an optimal safety profile since no patients reported any side or adverse events of any grade after the intravenous (i.v.) administration of the radiopharmaceutical, thus confirming that ^99m^Tc-IL2 can be used without any special precautions in humans [152]. Moreover, this modality resulted in being able to discriminate between true-progression (bad prognosis) and pseudo-progression (good-prognosis).

### 7.5. Evaluation or Response to Immunotherapy

A major task for the imagers in future, is to develop criteria to evaluate response on immunotherapy, and preferably as early as possible to allow discontinuation of therapy in non-responders to avoid useless side effects and to keep the economic burden as low as possible. Nowadays, therapy response is based on morphological criteria (RECIST criteria and immune-related response criteria (irRC), although RECIST criteria showed some limitations [153]; sometimes PET response criteria in solid tumours (PERCIST) or European Organization for Research and Treatment of Cancer (EORTC) criteria are used to assess the metabolic response to therapy by ^18^F-FDG-PET [106]. From early trials of immune-based therapy in melanoma, however, it became clear that sometimes unique response patterns exist, called pseudo-progression. Some patients whose disease met the criteria for disease progression based on RECIST were noted to have late but durable responses [154,155]. Therefore, recently a consensus guideline, called iRECIST, was developed for the use of RECIST in cancer immunotherapy trials, to ensure consistent design and data collection and to facilitate the on-going collection of trial data [156].

^18^F-FDG-PET may not be the best method for early response monitoring in immunotherapy, since ^18^F-FDG is not only taken up in tumour cells but also in inflammatory cells. Immunotherapeutic drugs also evoke an inflammatory response, with the consequence that ^18^F-FDG-PET may show an increase of metabolic burden as a result of pseudo-progression. Only a few example studies on this topic are available, but all show the inability of ^18^F-FDG-PET in the early phase after the start of the immunotherapy to differentiate between pseudo-progression (patients that should continue with therapy) and real progressive disease (a patient whose therapy should stop) [157,158]. Imaging specialist have to make huge efforts to define more accurately response to immunotherapy, by defining strict criteria with ^18^F-FDG-PET, or by using other more specific tracers targeting the immune system and so directly visualizing the immune reaction. In this view, from published studies, radiolabelled IL2 represents a promising tool able to differentiate pseudo-progression and real tumour progression thus providing important information for therapy decision making [110,152].

## 8. Conclusions

In this review, we have summarized several different radiopharmaceuticals and their use for imaging in different diseases (Table 1).

Medical research and imaging have progressively adopted the strategy to move from the concept of the organism intended as whole, to a molecular one in order to really understand the biological basis of diseases. As a consequence, diagnostic imaging is deeply connected to personalized medicine by finding new ways to predict the efficacy of a drug and personalize treatment. In some cases, as for RA, this concept goes further ahead to a “lesion-based therapy” rather than “person-based therapy”. Indeed, by studying patients with several radiopharmaceuticals, targeting different biological pathways, it appeared clear that lesions may have a different biology and evolution, thus requiring a different therapeutic approach. The same may apply to cancer metastases.

To this aim, in recent decades several efforts have been made in nuclear medicine. It is now possible to study in vitro, and in some circumstances in vivo too, many molecules that play a central role in the development of infectious, inflammatory and oncological diseases. Understanding the real pathogenesis and cellular network of these conditions is mandatory to finally reach an individualised therapy that takes into account the expression of specific inflammatory targets in order to select patients for the best therapeutic option. By assessing which molecules are expressed at the disease site and quantifying their presence, it will be possible to choose the most suitable therapy for the individual patient and to predict its efficacy. This kind of approach could provide an indispensable tool for therapy decision making avoiding unnecessary or useless drugs. This could be ideally applied to all the infective/inflammatory or neoplastic diseases mentioned above and many other disorders. Since many therapies, especially with MoAbs, are very expensive, molecular imaging techniques may also lead to cost-effectiveness by helping in decision making about which therapeutic agent is suitable for the individual patient.

More studies with more representative sample size are necessary and new and more specific molecules must be investigated in order to really define the clinical impact of these techniques and to shift from an empirical therapy to a personalized one.

## Figures and Tables

**Table 1:** Overview of discussed radiopharmaceuticals.

	Radiopharmaceutical	Indication
**Infectious diseases**	^99m^Tc/(^111^In)-WBC	Infection
	^99m^Tc-besilesomab (Scintimun)	Infection
	^18^F-FDG	Infection, inflammation, oncology
	^99m^Tc-ciprofloxacin (Infecton^®^)	Bacterial infection
	^99m^Tc-ubiquicidin	Bacterial infection
	^99m^Tc-fluconazole	Fungal infection
	^18^F-FEAU	Herpes Simplex Virus
**Inflammatory diseases**	^99m^Tc/^123^I/^18^F-IL2	Inflammatory bowel disease, Sjögren Syndrome, type 1 diabetes, thyroiditis, inflammatory plaque, rheumatoid arthritis
	^111^In/^68^Ga-somatostatin analogues	Rheumatoid arthritis
	^99m^Tc/^111^In/^123^I-anti-TNFα MoAb (infliximab, adalimumab, golimumab, certolizumab pegol, etanercept)	Rheumatoid arthritis
	^99m^Tc/^111^In/^89^Zr-rituximab^®^	Rheumatoid arthritis
**Tumour environment**	^99m^Tc/^111^In/^89^Zr-bevacizumab	Breast cancer, renal cell carcinoma
	^99m^Tc/^123^I/^18^F-IL2	Melanoma, squamous cell carcinoma of head and neck, renal cell carcinoma
	^111^In/^89^Zr/^64^Cu-PD1/PDL-1 MoAb (pembrolizumab, nivolumab, atezolizumab)	Metastatic lung cancer, bladder cancer, urothelial carcinoma, melanoma
	^18^F/^89^Zr/^64^Cu-CTLA-4 MoAbs (ipilimumab, tremelimumab)	Melanoma, colon cancer

WBC = white blood cells; (^18^F)-FDG = fluorodeoxyglucose, (^18^F)-FEAU = fluoro-5-ethyl-1-beta-D-arabinofuranosyluracil; IL2 = interleukin-2; TNFα = tumor necrosis factor α; MoAb = monoclonal antibody, PD1/PDL1 = programmed cell death protein 1/programmed cell death protein ligand 1.
